# Global MicroRNA Expression Profiling of Buffalo (*Bubalus bubalis*) Embryos at Different Developmental Stages Produced by Somatic Cell Nuclear Transfer and In-Vitro Fertilization Using RNA Sequencing

**DOI:** 10.3390/genes13030453

**Published:** 2022-03-01

**Authors:** Pallavi Goel, Shivani Malpotra, Songyukta Shyam, Deepak Kumar, Manoj Kumar Singh, Prabhat Palta

**Affiliations:** Embryo Biotechnology Lab, Animal Biotechnology Centre, ICAR-National Dairy Research Institute, Deemed University, Karnal 132001, Haryana, India; malpotras@gmail.com (S.M.); songyukta999@gmail.com (S.S.); deepakkumar1445@gmail.com (D.K.); drmanojvet@gmail.com (M.K.S.); prabhatpalta@yahoo.com (P.P.)

**Keywords:** somatic cell nuclear transfer (SCNT), miRNAs, In-vitro fertilization (IVF), bovine, RNA sequencing, gene ontology

## Abstract

Despite the success of cloning technology in the production of offspring across several species, its application on a wide scale is severely limited by the very low offspring rate obtained with cloned embryos. The expression profile of microRNAs (miRNAs) in cloned embryos throughout embryonic development is reported to deviate from regular patterns. The present study is aimed at determining the dynamics of the global expression of miRNA profile in cloned and in-vitro fertilization (IVF) pre-implantation embryos at different developmental stages, i.e., the two-cell, eight-cell, and blastocyst stages, using next-generation sequencing. The results of this study suggest that there is a profound difference in global miRNA profile between cloned and IVF embryos. These differences are manifested throughout the course of embryonic development. The cloned embryos differ from their IVF counterparts in enriched Gene Ontology (GO) terms of biological process, molecular function, cellular component, and protein class categories in terms of the targets of differentially expressed miRNAs. The major pathways related to embryonic development, such as the Wnt signaling pathway, the apoptosis signaling pathway, the FGF signaling pathway, the p53 pathway, etc., were found to be affected in cloned relative to IVF embryos. Overall, these data reveal the distinct miRNA profile of cloned relative to IVF embryos, suggesting that the molecules or pathways affected may play an important role in cloned embryo development.

## 1. Introduction

Cloning by somatic cell nuclear transfer (SCNT) is one of the most important reproductive technologies. It can enable the multiplication of elite animals at a pace much faster than conventional breeding programs. SCNT allows for the rapid multiplication of animals carrying specific alleles for higher milk yield, better disease resistance, neater meat quality, and higher reproductive potential. Moreover, this technique can be used for the addition of desirable traits into the breeding population. SCNT is also an integral part of several other allied reproductive technologies, such as transgenesis, xenotransplantation, therapeutic cloning, disease modeling, etc. [1]. It has also been used for the conservation and restoration of endangered species [2,3]. The birth of the first cloned mammal, ‘Dolly’, a sheep successfully cloned through SCNT in 1996, was a watershed moment in the history of cloning [4]. SCNT has since been efficaciously employed for the cloning of various other mammalian species [5,6].

Although successful live births have been achieved in over 20 mammalian species through SCNT, this cloning technology suffers from the serious problem of very low live birth rates with nuclear transferred (NT) embryos [5,6,7,8]. Since efforts to address this issue have not been fully successful, cloning by SCNT has not yet been applied on a large scale. In comparison to the live birth rate of over 40% obtained with bovine IVF embryos [9], the corresponding figure for NT embryos is only 9% [10,11] to 5–20% [6,12]. Across species, the live birth rate obtained with NT embryos is only 1–6% [3,8,12].

In SCNT, the genetic material of a somatic cell is transferred to an enucleated oocyte. The differentiated nucleus of a somatic cell is subjected to de-differentiation to reach the stage of totipotency. This process of making a differentiated cell totipotent is known as nuclear reprogramming. The resultant totipotent embryo is genetically identical to the donor somatic cell. The primary reason for low cloning efficiency is believed to be incomplete nuclear reprogramming, i.e., the process of reversing a differentiated somatic nucleus to a totipotent embryonic state after nuclear transfer [13,14]. Nuclear reprogramming essentially involves the abolishing of the expression profile of a differentiated cell and the establishment of a new embryo-specific expression. This dramatic change in the gene expression profile involves 10,000 to 12,000 genes which drive embryonic and fetal development [13]. 

The aberrant gene expression in cloned embryos as a consequence of incomplete nuclear reprogramming may be partly due to alteration in the expression profile of microRNAs (miRNAs). miRNAs are a class of small non-coding RNAs, ~22 nucleotides in length, which are one of the most important molecules involved in the regulation of gene expression generally; one gene can be regulated by several miRNAs, and one miRNA can regulate the expression of several target genes [15]. miRNAs bind to the 3′ untranslated region of mRNAs and control target gene expression either by translational repression or by mRNA degradation. miRNAs are an important component of epigenetic changes. For example, epi-miRNAs directly target epigenetic enzymes or functional protein complexes and thus play an important role in the regulation of DNA methylation or histone modifications [16,17]. miRNAs are also implicated in the regulation of cellular reprogramming through genes involved in epigenetic reprogramming and pluripotency [18]. 

miRNAs are involved in many biological processes, including proliferation, differentiation, gene expression regulation, oogenesis [19], and spermatogenesis [20]. miRNAs are present in human [21,22,23], bovine [24], and murine [25,26] gametes and pre-implantation embryos, where they play crucial roles in various pathways involved in embryo development. miRNAs are involved in the regulation of many important events in reproduction, such as oocyte maturation and fertilization [27], embryo development [28,29,30], and maternal-to-embryonic transition [31]. 

Since their discovery, miRNAs have been among the most extensively studied molecules. However, our knowledge about miRNA expression during mammalian pre-implantation embryo development and its role in the reprogramming of cloned embryos is still very limited. Several studies have been conducted on the expression analysis of a few selected miRNAs in IVF embryos using methods that can identify only a small number of miRNAs [32,33,34]. A few studies have also been conducted at the whole genome level using microarrays in cloned and IVF embryos at a specific stage of development [30,35]. Elucidating the global miRNA expression profile of NT embryos and understanding how it differs from that of IVF embryos could help in identifying the abnormalities in the NT embryos and eventually improve their quality to increase the live birth rate. To date, there is no report on the global miRNA expression profile of cloned embryos and its comparison with that of their IVF counterparts at different developmental stages in any species. The present study was, therefore, carried out to study the dynamic of the global expression of miRNA profile in cloned and IVF pre-implantation embryos at different developmental stages using next-generation sequencing. In the process, the associated networks and pathways in pre-implantation embryo development were predicted and the important differentially expressed miRNAs in cloned and IVF embryos were validated by real-time PCR.

## 2. Materials and Methods

All the chemicals and media were purchased from Sigma-Aldrich Corp. (St. Louis, MO, USA), and all the plasticware was purchased from Becton, Dickinson, and Co. (Lincoln Park, NJ, USA) or Nunc (Roskilde, Denmark) unless otherwise mentioned. Fetal bovine serum (FBS) was obtained from Gibco Life Technologies (Gaithersburg, MD, USA), whereas Research Vitro Cleave medium (K-RVCL) was purchased from William A. Cook (Brisbane, Australia). 

### 2.1. Production of Pre-Implantation Embryos by Hand-Made Cloning (HMC) 

The useable quality of COCs obtained from abattoir buffalo ovaries based on their morphology and BCB treatment was matured with the IVM medium (TCM-199 + 10% FBS + 5 µg/mL FSH-p + 1 μg/mL estradiol-17β + 0.81 mM sodium pyruvate + 50 µg/mL gentamicin sulfate). Tail skin fibroblast cells from a buffalo bull (Mu-4093) from CIRB, Hisar, Haryana, India, were used as donor cells for HMC, which were established and characterized by the standard protocol [36]. The cells were synchronized in the G1 stage of the cell cycle by growing them in culture to full confluence for contact inhibition, as described previously [37]. HMC, which included IVM, cumulus/zona removal, manual enucleation, fusion, activation, and in vitro culture (IVC), was performed as described earlier [38]. After the in-vitro culture, the pool of embryos at each developmental stage (two-cell, eight-cell, and blastocyst) was collected in triplicates after 14–16 h_,_ 36–40 h, and at Day 8, respectively. Each pool of cloned two-cell, eight-cell, and blastocyst stage embryos consisting of 500, 315, and 30, respectively, were used for RNA-seq. The blastocyst rate for the cloned embryos varied from 30–35%, whereas the cleavage rate for the two-cell and eight-cell developmental stages was recorded as 95–98% and 70–80%, respectively. The embryos were washed three times with DEPC-treated water and stored at −196 °C in liquid nitrogen.

### 2.2. Production of Pre-Implantation Embryos by IVF

For the IVF embryos, usable quality COCs obtained from the ovaries of slaughtered buffaloes were subjected to BCB staining to obtain COCs with high developmental competence. BCB+ oocytes were subjected to IVM, IVF, and IVC, as described previously [39].

For IVF, tail fibroblast cells of semen from the same bull (Mu-4093) were used as nuclear donor cells for HMC to produce genetically half-identical embryos in order to minimize the genetic variability between the embryos produced by the two approaches. After in vitro culture, the pool of embryos at the two-cell, eight-cell, and blastocyst stages was collected in triplicates after 24–26 h, 68–72 h, and at Day 8, respectively. An identical number of embryos as mentioned above was produced from the IVF used for RNA-seq. Likewise, for IVF embryos, the blastocyst rate was observed to vary from the 10–15% rate, and a cleavage rate of 15–22% was observed for the two-cell and eight-cell stages. The embryos were washed with DEPC-treated water and subsequently stored at −196 °C in liquid nitrogen. 

### 2.3. Extraction of Total RNA for Small-RNA Sequencing

Total RNA was isolated from the respective pools of cloned and in vitro-fertilized embryos using the Single Cell RNA Purification Kit (NORGEN, Thorold, ON, Canada) as per the manufacturer’s instructions. Quantification of RNA and its purity was analyzed through qubit reads. The RNA yield varied between 100 and 150 ng for each pool, and the 260/280 value was found to be between 1.79 and 1.90. Library preparation, sequencing, and data analysis were carried out by a commercial sequencing service provider (DNA Xperts, New Delhi, India). The quality of RNA isolated for sequencing from biological replicates (*n* = 3 each) of cloned (two-cell, eight-cell, and blastocyst) and IVF (two-cell, eight-cell, and blastocyst) embryos was examined on Agilent 2100 Bioanalyzer (Agilent Technologies, Santa Clara, CA, USA) using the Agilent Total RNA 6000 pico series kit. RNA samples with RIN values ranging from 6 to 10 were used further for the library preparation step.

### 2.4. cDNA Library Preparation and Sequencing

In accordance with the manufacturer’s instructions, QIAseq miRNA Library Kit (Thermo Fisher Scientific NGS Systems) was used for cDNA library preparation by taking the equimolar molar amount of RNA from cloned and IVF two-cell, eight-cell, and blastocyst stage embryos. The library was prepared in the single-end 1 × 50 bp format with an average fragment length of approximately 150 bp. The Agilent DNA High Sensitivity 1000 kit was employed for the quality control analysis of the library. An Illumina HiSeq 2500 instrument was used for the sequencing run. A quality check (QC) of the raw single-end reads was carried out using FastQC. All the reads were found to be of good quality based upon the Phred score values, which were greater than 20. 

### 2.5. miRNome Data Analysis

The UEA small RNA workbench (version 4.5) software was used to analyze the raw data. The clean reads were aligned to the Bos Taurus reference genome, UMD 3.1.1. The known miRNAs were identified using miRProf in miRBase 22.1, and the novel miRNAs were identified using the miRCat software. The miRNome data analysis workflow employed in the present study consisted of the following major steps, viz., raw data mapping onto the reference genome, sequence of Bos Taurus, number of mapped reads to each known miRNA calculation by miRProf, raw and normalized read counts obtained as output of miRProf, loading of read counts data into the DESeq2 software, normalization of data, identification of differentially expressed miRNAs based upon fold change and P-adjusted value criteria, and classification/clustering of genes. The Gene Ontology enrichment analysis was performed using the PANTHER classification system. Target genes were identified using the TagetScan software. Detailed pathway analysis was conducted using the Kyoto Encyclopedia of Genes and Genome (KEGG).

### 2.6. Validation of RNA-seq Data by qPCR

RNA was isolated from the three biological replicates of cloned and IVF two-cell, eight-cell, and blastocyst stages consisting of 600, 270, and 90 embryos, respectively, for qRT-PCR analysis as described above. RNA purity for each pool was determined by the A260/280 ratio for each replicate. The sample with a ratio between 1.9 and 2.1 was considered for qRT-PCR. For miRNA expression analysis, cDNA preparation was performed using the miRCURY LNA RT kit (Qiagen, MD, USA) according to the manufacturer’s instructions. A total of 5 ng/μL of template RNA was used for cDNA preparation followed by the incubation of the reaction mixture for 60 min at 42 °C. Heat inactivation of reverse transcriptase was performed for 5 min at 95 °C. miRNA cDNA was then immediately cooled to 4 °C and stored at 20 °C for further use. For miRNA qPCR analysis, locked nucleic acid (LNA™)-based universal primers were used (Qiagen, MD, USA) (Table S1).

## 3. Results

### 3.1. miRNA Profiling in Bovine Embryos

**Figure 1:**
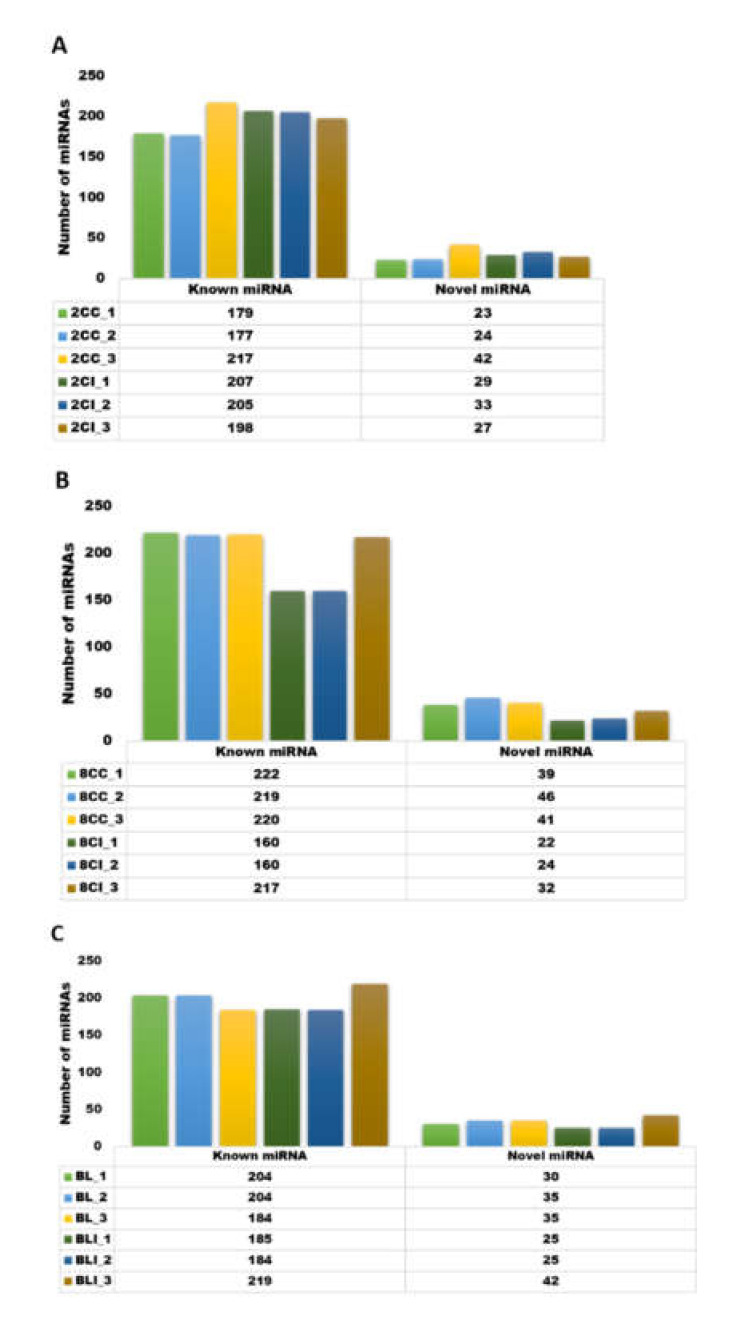
Number of known and novel miRNAs in the three replicates of (**A**) 2-cell stage cloned (2CC_1, 2CC_2, and 2CC_3) and 2-cell IVF embryos (2CI_1, 2CI_2, and 2CI_3); (**B**) 8-cell stage cloned (8-CC_1, 8CC_2, and 8CC_3) and IVF embryos (8CI_1, 8CI_2, and 8CI_3); and (**C**) cloned blastocyst (BL_1, BL_2, and BL_3) and IVF blastocyst (BLI_1, BLI_2, and BLI_3).

**Figure 2:**
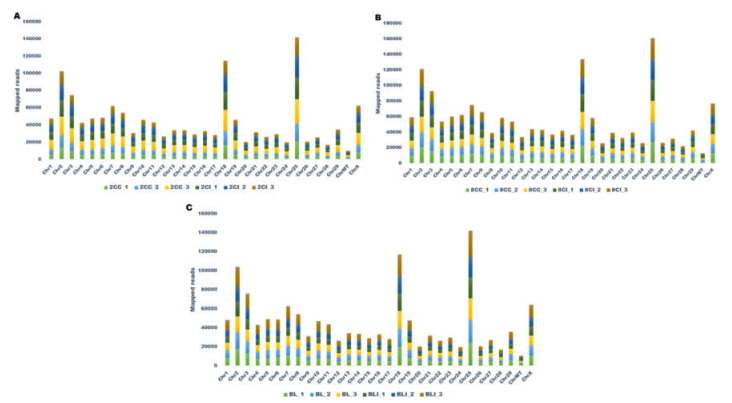
Reads of (**A**) 2-cell stage cloned and IVF embryos (2CC_1, 2CC_2, 2CC_3, 2CI_1, 2CI_2, and 2CI_3), (**B**) 8-cell stage cloned and IVF embryos (8CC_1, 8CC_2, 8CC_3, 8CI_1, 8CI_2, and 8CI_3), and (**C**) cloned and IVF blastocyst (BL1, BL2, BL3, BLI_1, BLI_2, and BLI_3) were mapped to different chromosomes of *Bos Tauras* (reference genome-UMI 3.1.1). The maximum number of reads was found to map on chromosome number 25.

**Figure 3:**
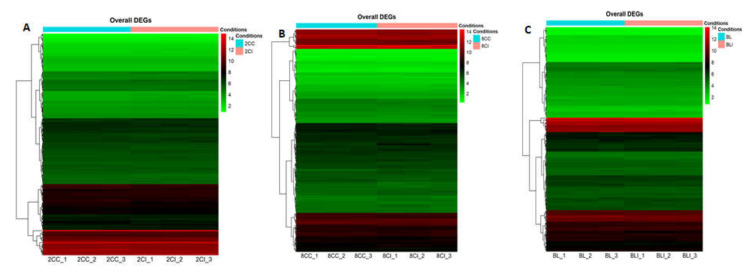
Hierarchical clustering analysis of miRNAs expressed differentially in cloned and IVF embryos based upon RPKM value, reflecting similar expression patterns between replicates of the same origin. (**A**) For 2-cell stage embryos, cloned replicates were named 2CC_1, 2CC_2, and 2CC_3. Similarly, for IVF replicates, the names were 2CI_1, 2CI_2, and 2CI_3. (**B**) For 8-cell stage embryos, the cloned replicates were named 8CC_1, 8CC_2, and 8CC_3. Similarly, for IVF replicates, the names were 8CI_1, 8CI_2, and 8CI_3. (**C**) For the blastocyst stage, the cloned replicates were named BL_1, BL_2, and BL_3. Similarly, for IVF replicates, the names were BLI_1, BLI_2, and BLI_3.

In the present study, we generated approximately 350GB of raw data to identify the population of miRNAs present in cloned and IVF embryos at different developmental stages (two-cell, eight-cell, and blastocysts) of buffalo. For all the samples, 79.67 to 85.40% of the total reads were aligned against the reference genome of *Bos taurus*, UMD 3.1.1. Detailed parameters of alignment at each developmental stage with three replicates are mentioned in Table 1, and a bar graph representation of the read alignment is shown in Figure S1. The raw reads and reads after the removal of the adapter (distinct reads) are shown in Table 1. The number of known and novel miRNAs varied among the three biological replicates of cloned and IVF embryos at each stage (Figure 1). The raw reads were normalized, and the normalized signal values were used for subsequent data analysis. The box-whisker plot showing the distribution of normalized signal values in all the replicates of cloned and IVF embryos at different stages is presented in Figure S2. The quality of the sequencing data generated was analyzed by principal component analysis (PCA), which shows that the two-cell, eight-cell, and blastocyst stage embryos overall were grouped according to their origins (Figure S3). The cloned replicates were clustered together in one group while the IVF replicates were together in another group. The reads obtained were aligned on different chromosomes and the maximum number of reads was found to map on chromosome number 25 for all the three stages (Figure 2). Overall heat maps consisting of all the three biological replicates of the two-cell, eight-cell, and blastocysts stage of cloned and IVF embryos were generated using normalized values, i.e., reads per kilobase million (RPKM) values. (Figure 3). The heat map showing a clear disparity among the expression patterns among the two groups, i.e., cloned and IVF embryos at each stage, is shown in Figure S4.

### 3.2. Identification of Differentially Expressed miRNAs in Pre-Implantation Embryos

**Figure 4:**
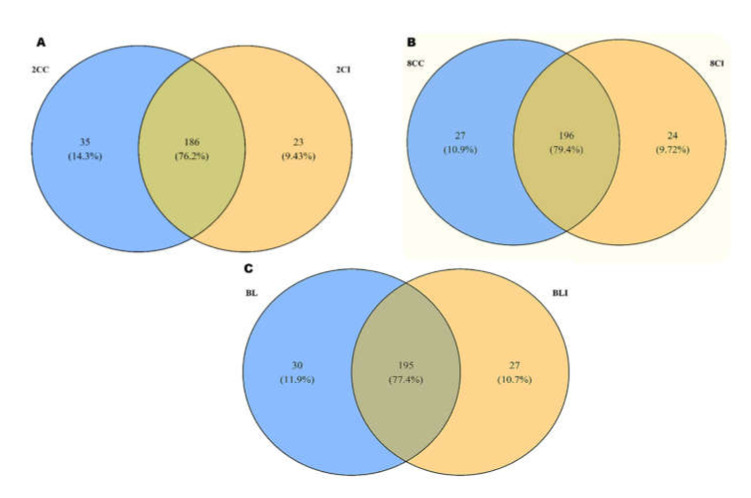
Venn diagram showing the overall commonly and uniquely expressed miRNAs in cloned and IVF (**A**) 2-cell (2CC and 2CI), (**B**) 8-cell (8CC and 8CI), and (**C**) blastocyst-stage (BL and BLI) embryos.

**Figure 5:**
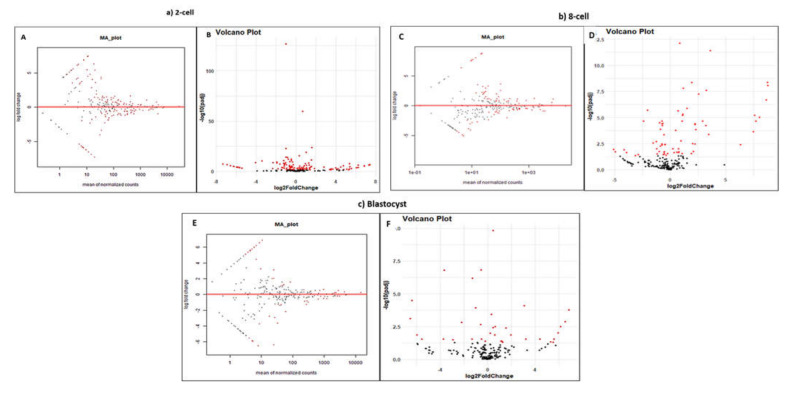
(**A**) MA plot depicting the expression pattern of up- and down-regulated miRNAs in cloned relative to IVF embryos at the 2-cell stage. The Y-axis represents the log fold ratio (M) and the X-axis is the mean average of normalized counts. The red dots represent differentially expressed miRNAs having adjusted p-values above the threshold value, whereas the black dots represent differentially expressed miRNA having p-values below the threshold. (**B**) Volcano plot showing differentially expressed up- and down-regulated miRNAs in cloned relative to IVF embryos at the 2-cell stage. The red dots indicate miRNAs significantly differentially expressed, whereas the black dots indicate miRNAs the expression of which was non-significant in the two groups. The dots towards the left, right, and top denote down-regulated, up-regulated, and most significantly expressed miRNAs, respectively. (**C**,**D**) MA plot and volcano plot for 8-cell stage cloned embryos relative to their IVF counterparts. (**E**,**F**) MA plot and volcano plot for blastocyst stage cloned embryos relative to their IVF counterparts.

A total of 244, 247, and 252 miRNAs were found to be differentially expressed at the two-cell, eight-cell, and blastocyst stages in cloned relative to IVF embryos, respectively. In the two-cell stage, out of 244 miRNAs, 186 were commonly expressed in both cloned and IVF two-cell stage embryos, 35 miRNAs were expressed exclusively in cloned embryos, whereas 23 were expressed exclusively in IVF embryos. Similarly, at the eight-cell stage, 196 miRNAs were commonly expressed in both cloned and IVF embryos, 27 miRNAs were expressed exclusively in cloned embryos, whereas 24 were expressed exclusively in IVF embryos. Moreover, at the blastocyst stage, 195 were commonly expressed in both cloned and IVF blastocysts, 30 miRNAs were expressed exclusively in cloned embryos, whereas 27 were expressed exclusively in IVF embryos (Figure 4). Down-regulated, top 30 differentially expressed miRNAs with fold change (FC) ≥ 2 at all three developmental stages of cloned embryos with respect to their IVF counterparts are given in Tables S2–S4. In all the three comparisons, miRNAs unique to the cloned group were down-regulated, whereas miRNAs unique to IVF groups were up-regulated. MA and volcano plots were also used to visualize the distribution pattern of differentially expressed miRNAs in cloned embryos with respect to IVF preimplantation embryos. Different types of distribution patterns were observed in all the developmental stages of the embryo (Figure 5).

**Figure 6:**
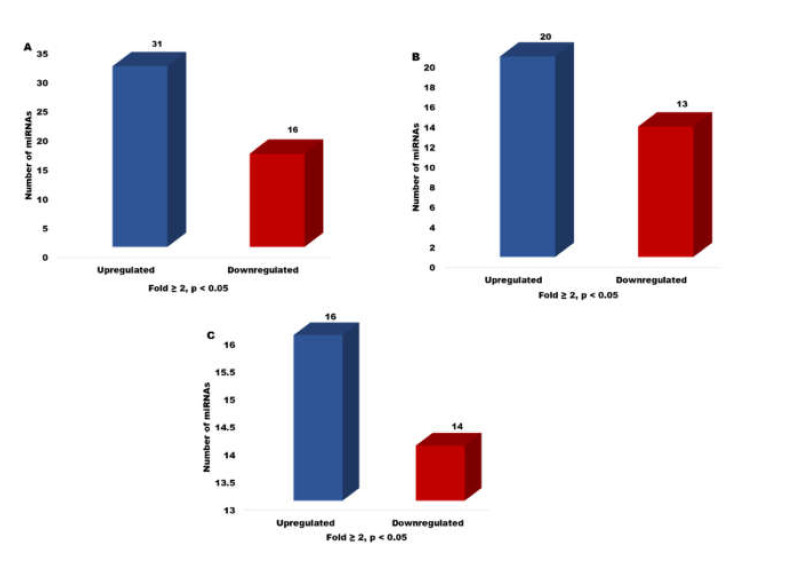
Bar graph depicting the number of differentially expressed miRNAs up- or down-regulated in cloned relative to IVF (**A**) 2-cell stage, (**B**) 8-cell stage, and (**C**) blastocyst stage embryos at FC ≥ 2 (*p* < 0.05).

In the present study, differential expression was also determined in terms of fold change values for all three stages of embryo development. The total number of differentially expressed miRNAs at different FC values (≥2 to <3-, ≥3 to <5-, and ≥5-folds) in the two-cell, eight-cell, and blastocysts stage is given in Figure S5. Furthermore, at different FC values, the number of up- and down-regulated miRNAs in cloned relative to IVF embryos for all three developmental stages is shown in Figure S6. At FC ≥ 2 (*p* < 0.05), 47 miRNAs were found to be differentially expressed in cloned relative to IVF two-cell stage embryos, out of which 31 were up-regulated and 16 were down-regulated. By contrast, in the eight-cell stage, 33 miRNAs were found to be expressed at a significant level (*p* ≤ 0.05) at FC ≥ 2 and 20 were up-regulated, whereas 13 were down-regulated. In the case of cloned blastocysts relative to IVF, 30 miRNAs were found to be differentially expressed, out of which 16 were up-regulated and 14 were down-regulated at FC ≥ 2 (*p* < 0.05) (Figure 6).

### 3.3. Identification of Commonly and Uniquely Expressed miRNAs

In the present study, we extrapolated the data from Figure S4 to find out the commonly and uniquely expressed miRNAs in cloned and IVF embryos at different FC levels. A minimum cut-off of two-fold change revealed that 66 miRNAs were expressed differentially, out of which 18 were unique to cloned embryos, 23 were unique to IVF embryos, and 25 were expressed in both types of two-cell stage embryos. Similarly, in the eight-cell stage, at a minimum cut-off of two-fold change, it was revealed that 76 miRNAs were expressed differentially, out of which 25 were unique to the cloned embryos, 24 were unique to IVF embryos, and 27 were expressed in both types of embryos. Moreover, in the case of blastocysts, at FC ≥ 2, 73 miRNAs were expressed differentially, out of which 30 were unique to the cloned blastocysts, 22 were unique to IVF blastocysts, and 21 were expressed in both types of blastocysts. From among the commonly expressed miRNAs, those which were up-and down-regulated at different fold change levels in cloned relative to IVF embryos are presented in Figure S7.

**Figure 7:**
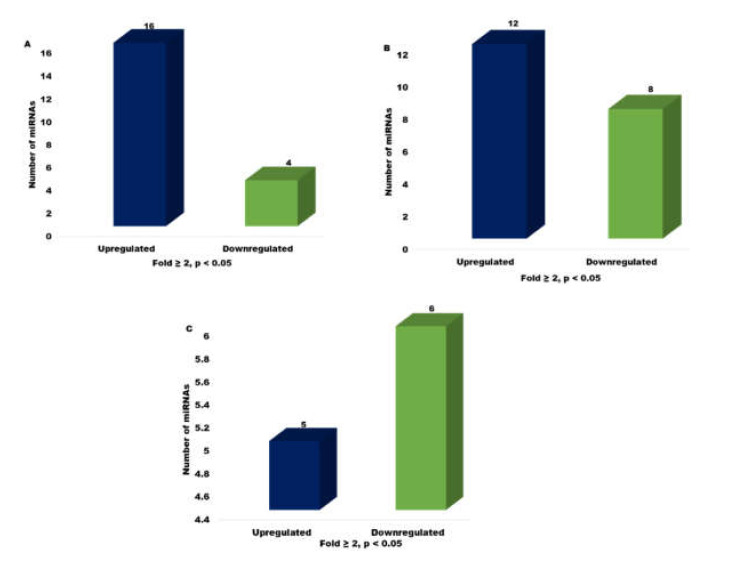
Bar graph depicting the number of commonly expressed miRNAs up- or down-regulated in cloned relative to IVF (**A**) 2-cell stage, (**B**) 8-cell stage, and (**C**) blastocyst stage embryos at FC ≥ 2 (*p* < 0.05).

At FC ≥ 2 and a significance level of *p* < 0.05, a total of 20 miRNAs were found to be commonly expressed between the two types of embryos, out of which 4 were down-regulated and 16 were up-regulated in cloned relative to IVF two-cell stage embryos. Whereas, in the eight-cell stage, out of 20 commonly expressed miRNAs in both types of embryos 8 were down-regulated and 12 were up-regulated in cloned relative to IVF embryos. A total of 11 miRNAs were found to be commonly expressed between the two types of blastocysts at a significance level of *p* < 0.05, out of which 6 were down-regulated and 5 were up-regulated in cloned relative to IVF blastocysts (Figure 7).

### 3.4. Gene Ontology and KEGG Analysis of Cloned vs. IVF Preimplantation Embryos

Using the PANTHER classification system, Gene Ontology (GO) analysis was performed in order to inspect the biological significance, detailed annotation of gene function, biological process, and cellular distribution of the targets of miRNAs expressed differentially between cloned and IVF embryos. Using GO terms, the NGS results were summarized to provide insights into the changes in miRNA expression between cloned and IVF embryos at the two-cell, eight-cell, and blastocyst stages. In the present study, the targets of miRNAs expressed differentially at fold change ≥2 in cloned relative to IVF embryos were used for GO analysis. The categories most enriched under the biological process GO term were cellular process, metabolic process, regulation of the biological process, and response to stimuli. Similarly, in the molecular function GO term categories, the most affected were binding activity and catalytic activity. In the case of the cellular component GO term, the cell part, cell, and organelle were found to be the most enriched. In the case of the protein class GO term, the metabolite interconversion enzyme, the protein modifying enzyme, the nucleic acid-binding protein, and the gene-specific transcriptional regulator were the most affected. The complete list of biological pathways, molecular functions, and cellular components detected in all three pre-implantation stages is shown in Table S5a–c.

### 3.5. Pathway Identification and Network Analysis

The KEGG pathway enrichment analysis helped us to demonstrate the relationship between the identified differentially expressed miRNA and their role in the developmental processes of the embryos. At the two-cell stage, 126 pathways were detected in cloned embryos relative to their IVF counterparts. Likewise, 122 pathways were detected in the eight-cell stage cloned embryos relative to their IVF counterparts. Moreover, 126 pathways were detected at blastocyst stage cloned embryos relative to their IVF counterparts. (The list of pathways found in all the three stages of embryo development are given in Table S6a–c.) Through KEGG analysis we identified the most significantly enriched pathway during the course of embryo development, which included apoptosis, cell cycle, MAPK signaling, mTOR, notch signaling, the p53 pathway, the PI3K-AKT signaling pathway, the RAS signaling pathway, signaling pathways regulating the pluripotency of stem cells, the TGF β signaling pathway, the ERBB signaling pathway, the Toll-like signaling pathway, and the Wnt signaling pathway. The notch signaling pathway was specifically enriched in two-cell stage cloned embryos relative to IVF embryos. 

KEGG analyses also revealed that the significantly enriched metabolic pathways during embryo development were pyrimidine metabolism (bta00240), purine metabolism (bta00230), fatty acid biosynthesis (bta00061), and glycolysis/gluconeogenesis (bta00010). 

**Figure 8:**
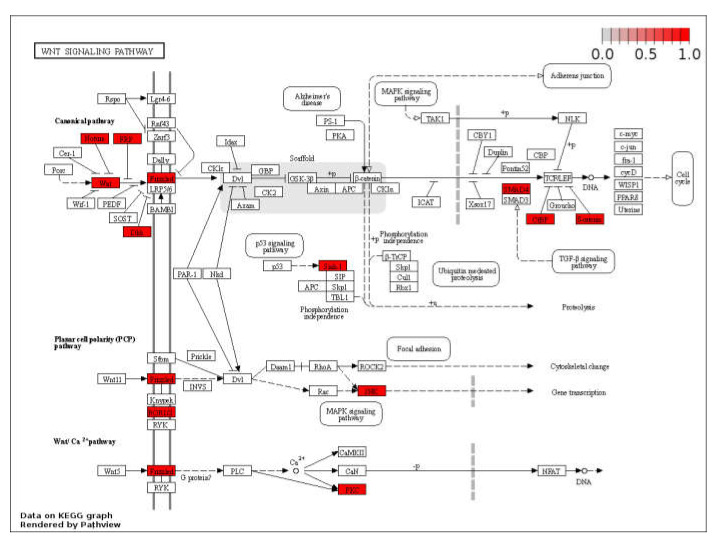
KEGG pathway analysis shows the Wnt signaling pathway and the target genes affected by under- and over-presented miRNAs in cloned relative to IVF embryos.

The Wnt signaling pathway is one of the most important signaling pathways during embryonic development. It plays a crucial role in regulating the development process of the blastocyst stage. In our study, the Wnt pathway was the most affected pathway during embryo development. With the help of KEGG analysis, we were also able to identify the target genes of up-regulated miRNA which were predominantly enriched in the Wnt pathway (Figure 8). The total number of genes involved in the Wnt signaling pathway and the number of genes affected in each stage are given in Table 2. miR-34, miR-345, and miR-409 were found to be up-regulated in cloned embryos at the two-cell stage and were predicted to regulate members of the Wnt signaling pathway such as *WNT2, WNT9A, WNT3A, WNT7A,* and *WNT7B*. Moreover, miR-331, miR-339, miR-342, and miR-345 were predicted to target *FZD2, FZD3, FZD5,* and *FZD7* at the two-cell stage. At the eight-cell stage, miR-486, miR-487, and miR-493 were also detected as targeting the *WNT7B* and *WNT9B* genes, whereas miR-338, miR-365, miR-371, and miR-378 were found to target *WNT3A, WNT9A, WNT7B,* and *WNT2*. Other miRNAs, such as miR-381, miR-455, miR-423, miR-93, and miR-574, were found to target the *DKK3, DKK4,* and *DKK1* genes, respectively.

### 3.6. Validation of RNA-seq Data

**Figure 9:**
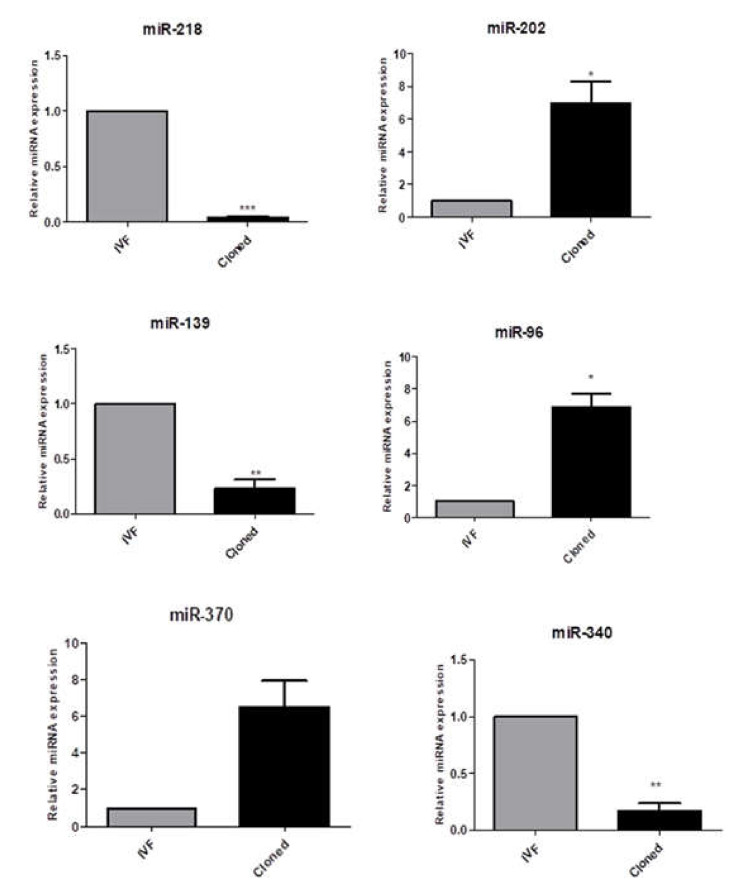
Validation of RNA-seq data by qPCR analysis of 6 miRNAs expressed differentially in cloned and IVF 2-cell, 8-cell, and blastocyst stage embryos. The pattern and magnitude of relative expression levels were found to be similar in RNA-seq and qPCR data. Bars with different superscripts differ significantly (* *p* < 0.05; ** *p* < 0.01; *** *p* < 0.0001). Values are mean ± SEM.

In order to validate the RNA-seq data, six miRNAs which were expressed differentially between cloned and IVF embryos at different developmental stages (two-cell, eight-cell, and blastocyst) were randomly selected based on their fold change for validation by qPCR analysis. These miRNAs were miR-218 (−5.64), miR-340 (−6.44), and miR-202 (6.02) from the two-cell stage, miR-96 (3.97), and miR-139 (−5.06) from the eight-cell stage, and miR-370 (4.42) from the blastocyst stage. All the selected miRNAs followed similar expression patterns in real-time PCR with respect to NGS data. The two housekeeping miRNAs, i.e., UniSp6 and miR-423, were used for the normalization of target miRNAs. The pattern and magnitude of relative expression levels were found to be similar in the RNA-seq and qPCR data (Figure 9).

## 4. Discussion

Somatic cell nuclear transfer is a potent artificial reproductive method that can be used to conserve key genetic features in animals, but it is also plagued by low offspring yield rates. This low efficiency is assumed to be caused by errors in donor cell reprogramming, which manifest as changes in epigenetic status and/or RNA abundance. Leveraging these errors for correction via molecular means, such as increasing expression of a key miRNA to maintain pluripotency, is one way to potentially improve the quality of SCNT embryos. miRNAs are small RNA molecules that are crucial for the correct expression of genes without becoming translated.

To date there is very little is known in regard to miRNAs and their potent role during embryonic development in mammalian species. There have been several reports on the expression analysis of a single or a few selected miRNAs in embryos. miRNA expression profiles undergo dynamic changes during pre-implantation embryo development, although significantly different miRNA profiles were observed with different methodologies [25]. However, these reports were not able to discover the miRNAs at the whole genome level by using techniques such as real-time PCR, microarray, etc. Cuthbert and co-workers examined the dynamic shifts in the profile of small non-coding RNAs in terms of their relative abundance during the maternal-to-embryonic transition period in the different developmental stages of SCNT and IVF embryos, i.e., two-cell, eight-cell, and morula, along with blastocyst-derived cells and donor cells used for cloning in cattle. They specifically found miR-2340, miR-345, and miR-34a to be differentially expressed at the morula stage and many other differentially expressed miRNAs in different developmental stages. Other than miRNA, they also profiled piRNA and tRNA in SCNT relative to IVF embryos [40]. To the best of our knowledge, a comparative global miRNA profile of cloned and IVF embryos using NGS has not yet been reported. Our group is the first to report the global miRNA profile in cloned embryos produced by the hand-guided cloning technique relative to IVF. Furthermore, our study provides deeper insight into the presence of miRNAs in cloned relative to IVF embryos during development. However, only three stages of buffalo in vitro embryos were covered under this study. miRNA profiling of in vivo counterparts of the embryos and donor somatic cells were not compared in the present report. The present study can be used as baseline data for future studies in embryos derived in vivo- to compare global miRNA profiles to understand how embryos derived in vitro deviate from those derived in vivo through the course of development. It is well known from previous studies that the miRNA expression of donor somatic cells shows significant changes upon nuclear reprogramming in hand-made cloning (HMC) embryos [35]. It would thus be of great interest to see these changes in donor somatic cells after reprogramming on a large scale using high-throughput techniques. Although this is the first study to represent the complete miRNA profile of buffalo embryos, owing to the involvement of a mixed population of male and female IVF embryos against exclusive male cloned embryos, these data may contain a systemic bias.

Lingenfelter et al., reported that miR181a was present in both bovine oocytes and bovine embryos; it had a high expression level in the early stages of development, which decreased to low levels at the blastocyst stage. It is thought to regulate nucleoplasmin2, a protein important in nuclear organization [41]. In this study, miR-181 followed the same trend, showing higher expression in the early stage and low expression in the blastocyst stage. In another study, the microRNA expression profile was analyzed in Day-17 elongated, cloned, in vitro-produced embryos (IVP), as well as the expression profile of the somatic cell, by using a microarray. A total of 39 miRNAs were found to be expressed in Day-17 embryo produced by SCNT, whereas in IVP embryos 32 miRNAs were found to be expressed. Conclusively, they reported the difference in the expression profile of miRNAs SCNT elongated embryos when compared with the donor cell and embryos produced in vitro [35]. Coutinho et al., also performed miRNAs profiling in bovine Day-30 embryos as well as in different tissues using a real-time PCR array, in which they identified 49 miRNAs as expressed in embryos [28]. By contrast, our study was one step ahead in terms of technique, and deciphered the miRNAs and analyzed their kinds, revealing a distinct pattern of miRNA expression in cloned relative to IVF embryos in all three stages. Interestingly, in our data, we also found some miRNAs present consistently in all three stages with the same regulation with variable degrees of expressions such as miR-378, miR-11988, miR-218, miR-210, and miR-371.

In addition, some of the miRNAs were found to be exclusive to a particular developmental stage, such as miR-6121 and miR-582, which were found to be exclusively expressed at the two-cell stage, showing their maternal origin. At the eight-cell stage, miR-377 and miR-14 were found to be uniquely expressed, showing that these miRNAs may have a role during the maternal-to-zygotic transition period. miR-33 and miR-100 were expressed exclusively in the blastocyst stage. These miRNAs may have a role in the implantation and further development of embryos.

In addition, we found that 22 categories were enriched under the protein class. Our results found in the present study that the major pathways related to embryonic development which were found to be affected in cloned blastocyst-stage embryos relative to their IVF counterparts were the apoptosis signaling pathway, the Wnt signaling pathway, the Ras pathway, the CCKR signaling map, the TGF-β signaling pathway, the PDGF signaling pathway, the integrin signaling pathway, the FGF signaling pathway, the cadherin signaling pathway, and the EGF receptor signaling pathway. It is evident that miRNAs, being potent regulatory molecules, play a crucial role in several signaling pathways [15]. A large variety of cellular processes such as cell proliferation, cell fate, cell–cell adhesion, pluripotency, and cellular polarity are regulated by Wnt signaling pathways [42,43,44]. It has been reported that genes from the Wnt signaling pathway play an essential role in the various biological processes involved in the development of follicles and oocytes, and also regulate embryo cleavage and implantation [45,46]. Denicol et al., reported a decrease in the development of bovine embryo development in the context of blastocyst rate and cell number affected due to activation of the Wnt signaling pathway after embryonic genome activation [47]. The author used Dickkopf-1 treatment to antagonize Wnt signaling, which significantly improved the survival of the embryo after transfer in the recipient. By contrast, Xie and co-workers observed no significant reduction in blastocyst rate, but observed a completely compromised implantation rate upon the silencing of the Wnt signaling pathway [48].

Moreover, another report showed that miR-320 in the follicular fluid of mice is associated with embryo development and is involved in the regulation of the Wnt signaling pathway, which is in agreement with our own data from the present study [49]. Results from previous studies from our lab are also in agreement with the data from our present study, viz., Sood et al., performed an RNA-seq study on cloned and IVF blastocysts in which most of the genes related to the Wnt signaling pathway were found to be up-regulated, whereas inhibitors such as DKK-1 were down-regulated, suggesting an altered expression of Wnt signaling pathways in SCNT blastocyst [50]. Likewise, Shyam et al., reported faulty Wnt signaling in SCNT embryos that was rectified with Dickkopf-1 (a Wnt signaling inhibitor) and CSF-1 treatment in in vitro culture media [51]. This treatment significantly improved the development competence, quality, and change in gene expression and live birth rate of buffalo embryos. Altogether these studies imply the importance of the Wnt signaling pathway during early embryo development and implantation. The number of genes affecting in the Wnt signaling pathway in SCNT embryos are shown in Figure 8.

In conclusion, the present study suggests that there is a profound difference in global miRNA profile between cloned and IVF embryos. These differences are manifested throughout embryonic development. Secondly, cloned embryos differ from their IVF counterparts in the enriched GO terms of biological process, molecular function, cellular component and protein class categories in terms of the targets of miRNAs expressed differentially between cloned and IVF embryos. Lastly, a large number of pathways are affected in cloned relative to IVF embryos. Among these, the major pathways related to embryonic development are the Wnt signaling pathway, the apoptosis signaling pathway, the FGF signaling pathway, the p53 pathway, etc. 

As a result, this report uncovers the miRNA populations linked to the development of cloned embryos relative to their IVF counterparts. In animals, these regulatory molecules could be crucial for zygotic genome activation and embryo development. Their role in cloned embryos should be examined further in future studies, as their function in mammalian embryogenesis is unclear. In addition, more studies are needed to determine the role of differently expressed miRNAs in modifying embryogenesis in cloned embryos, with a focus on the impact of these miRNAs on maternally generated transcripts and embryonic genome activation. This study can be extrapolated to find target genes affected by faulty microRNA regulation and use of epigenetic agents to improve blastocyst rate and the conception of cloned embryos. 

## Figures and Tables

**Table 1:** Alignment statistics of cloned and IVF embryos at different developmental stages.

Parameter	Total Number of Reads	Distinct Number of Reads	Aligned Reads (%)	Unaligned Reads (%)	Maximum Read Length
2CC-1	17,307,418	232,369	79.67	20.33	50
2CC-2	15,731,948	227,625	79.82	20.18	50
2CC-3	16,309,020	392,869	81.38	18.62	50
2CI-1	13,422,098	314,645	83.54	16.46	50
2CI-2	12,758,526	311,976	83.66	16.34	50
2CI-3	11,893,184	225,993	85.40	14.60	50
8CC-1	17,409,668	364,676	81.35	18.65	50
8CC-2	12,483,044	331,093	82.16	17.84	50
8CC-3	14,840,784	351,968	81.80	18.20	50
8CI-1	13,822,944	234,722	83.31	16.69	50
8CI-2	13,308,848	237,288	83.49	16.51	50
8CI-3	15,839,750	366,705	83.71	16.29	50
BL-1	12,608,590	285,577	85.59	14.41	50
BL-2	10,784,686	273,300	85.92	14.08	50
BL-3	10,875,612	276,876	84.20	15.80	50
BLI-1	12,380,724	250,071	82.35	17.65	50
BLI-2	12,573,252	252,929	82.39	17.61	50
BLI-3	14,146,486	355,853	83.23	16.77	50

**Table 2:** Genes annotated in the Wnt signaling pathway in cloned embryos relative to IVF embryos.

Stages	Total Genes	Annotated Genes in the Wnt Pathway	Log Fold Change	*p*-Value
2CC-vs-2CI	2276	31 (1.37%)	1.06	6.97 × 10^−5^
8CC-vs-8CI	1934	30 (1.55%)	1.24	2.45 × 10^−5^
BL-vs-BLI	2631	41 (1.56%)	1.22	2.37 × 10^−5^

## Data Availability

The author confirms that the data supporting the findings of this study are available within the article and Supplementary File. All details regarding the data could be requested from the corresponding author.

## Supplementary Materials

The following are available online at https://www.mdpi.com/article/10.3390/genes13030453/s1, Figure S1: High-quality reads of (a) 2-cell stage (b) 8-cell stage (c) blastocyst stage cloned and IVF embryos were aligned against Bos taurus reference genome, UMD 3.1.1. Figure S2: Box whisker plot showing the distribution of normalized signal values in the three replicates of (a) 2-cell stage, (b) 8-cell stage and (c) blastocyst stage cloned and IVF embryos. Figure S3: The quality of sequencing data generated was analysed by Principal Component Analysis (PCA). The 2D scatter plot shiows that the three replicated of (a) 2-cell, (b) 8-cell and (c) blastocyst stage cloned embryos clustered together in one group while the three replicated of (a) 2-cell, (b) 8-cell and (c) blastocysts stage IVF embryos clustered together in another group. A high correlation amongst the replicates of same origin was observed with correlation coefficient value of R = 1. Figure S4: Heat map of top 50 differentially expressed miRNAs at (a) 2-cell (2CC& 2CI), (b) 8-cell (8CC & 8CI) and Blastocyst (BL & BLI). Figure S5: miRNAs expressed differentially between cloned and IVF (a) 2-cell stage (b) 8-cell stage (c) blastocyst stage embryos were sorted on the basis of FC values. Figure S6: miRNAs expressed differentially between cloned and IVF IVF (a) 2-cell stage (b) 8-cell stage (c) blastocyst stage embryos at different FC values (≥2 to <3, ≥3 to <5 and ≥5-fold) were sorted on the basis of regulation. The graph represents the number of miRNAs up-or down-regulated in cloned compared to IVF embryos. Figure S7: miRNAs expressed commonly in cloned and IVF (a) 2-cell stage (b) 8-cell stage (c) blastocyst stage embryos at different FC values (≥2 to <3, ≥3 to <5 and ≥5-fold). The graph represents the number of miRNAs up-or down-regulated in cloned compared to IVF embryos. Table S1: Details of Locked nucleic acid (LNA) primers for miRNAs. Table S2: List of top 30 miRNAs upregulated and downregulated in cloned relative to IVF 2-cell stage embryos with minimum FC of ≥2. Table S3: List of top 30 miRNAs upregulated and downregulated in cloned relative to IVF 8-cell stage embryos with minimum FC of ≥2. Table S4: List of top 30 miRNAs upregulated and downregulated in cloned relative to IVF blastocyst stage embryos with minimum FC of ≥2. Table S5a: GO categories for Biological Process, Molecular Functions, Cellular and protein class enriched across the target genes of differentially expressed miRNAs in 2-cell stage cloned embryos relative to IVF counterparts. Total of 500 embryos in each replicate for both types of embryos were used (Biological Replicates, *n* = 3). Table S5b: GO categories for Biological Process, Molecular Functions, Cellular and protein class enriched across the target genes of differentially expressed miRNAs in 8-cell stage cloned embryos relative to IVF counterparts. A total of 315 embryos in each replicate for both types of embryos were used (Biological Replicates, *n* = 3). Table S5c: GO categories for Biological Process, Molecular Functions, Cellular and protein class enriched across the target genes of differentially expressed miRNAs in cloned blastocyst relative to IVF counterparts. A total of 30 blastocysts in each replicate for both types of embryos were used (Biological Replicates, *n* = 3). Table S6a: List of pathways found to be affected at 2-cell stage cloned embryos relative to IVF. Total of 500 embryos in each replicate for both types of embryos were used (Biological Replicates, *n* = 3). Table S6b: List of pathways found to affected at 8-cell stage cloned embryos relative to IVF. Total of 315 embryos in each replicate for both types of embryos were used (Biological Replicates, *n* = 3). Table S6c: List of pathways found to be affected in cloned blastocysts relative to IVF. Total of 30 blastocyst in each replicate for both types of embryos were used (Biological Replicates, *n* = 3).
